# An isoform of the plastid RNA polymerase-associated protein FSD3 negatively regulates chloroplast development

**DOI:** 10.1186/s12870-019-2128-9

**Published:** 2019-11-27

**Authors:** Sangyool Lee, Young Hee Joung, Ju-Kon Kim, Yang Do Choi, Geupil Jang

**Affiliations:** 1 School of Biological Sciences and Technology, Chonnam National University, Gwangju, 61186 Republic of Korea; 2 Graduate School of International Agricultural Technology and Crop Biotechnology Institute/Green BioScience and Technology, Seoul National University, Pyeongchang, 25354 Republic of Korea; 3 Department of Agricultural Biotechnology and Research Institute of Agriculture and Life Sciences, Seoul National University, Seoul, 08826 Republic of Korea; 4 The National Academy of Sciences, Seoul, 06579 Republic of Korea

**Keywords:** Chloroplast, Plastid-encoded RNA polymerase, PEP-associated protein, FSD3, FSD3S, Alternative splicing

## Abstract

**Background:**

Plastid-encoded RNA polymerase (PEP) plays an essential role in chloroplast development by governing the expression of genes involved in photosynthesis. At least 12 PEP-associated proteins (PAPs), including FSD3/PAP4, regulate PEP activity and chloroplast development by modulating formation of the PEP complex.

**Results:**

In this study, we identified *FSD3S*, a splicing variant of *FSD3*; the *FSD3* and *FSD3S* transcripts encode proteins with identical N-termini, but different C-termini. Characterization of FSD3 and FSD3S proteins showed that the C-terminal region of FSD3S contains a transmembrane domain, which promotes FSD3S localization to the chloroplast membrane but not to nucleoids, in contrast to FSD3, which localizes to the chloroplast nucleoid. We also found that overexpression of *FSD3S* negatively affects photosynthetic activity and chloroplast development by reducing expression of genes involved in photosynthesis. In addition, *FSD3S* failed to complement the chloroplast developmental defects in the *fsd3* mutant.

**Conclusion:**

These results suggest FSD3 and FSD3S, with their distinct localization patterns, have different functions in chloroplast development, and FSD3S negatively regulates expression of PEP-dependent chloroplast genes, and development of chloroplasts.

## Background

Chloroplasts have a unique genome and two distinct RNA polymerases, the nuclear-encoded RNA polymerase (NEP) and the plastid-encoded RNA polymerase (PEP), that mediate the transcription of plastid genes. NEP is a single-subunit RNA polymerase and PEP is a multimeric RNA polymerase composed of four core proteins, rpoA, rpoB, rpoC1, and rpoC2 [1–3]. Both NEP and PEP are required for chloroplast development [4, 5]. For example, the mutants with defects in *RPOTp* or *RPOTmp* encoding NEP show delayed chloroplast biogenesis and retarded growth, and the mutants lacking PEP activity display albino/ivory phenotypes [6–11]. It has been assumed that NEP functions at the beginning of chloroplast biogenesis and PEP functions in mature chloroplasts, based on the distinct activity between NEP and PEP; NEP is responsible for the expression of *rpoB* and other housekeeping genes*,* and PEP is responsible for the expression of photosynthesis-related genes. However, many studies using tobacco (*Nicotiana tabacum*) or barley (*Hordeum vulgare*) mutants lacking PEP activity showed that both NEP and PEP are active during all stages of chloroplast development [4, 5, 7].

PEP activity is essential for the formation of fully active chloroplasts, as it promotes the expression of photosynthesis-related genes [3]. PEP forms a complex with PEP-associated proteins (PAPs), and the *Arabidopsis thaliana* nuclear genome contains at least 12 *PAP* genes [3, 12], and all PAPs have also been identified in the nucleoid or transcriptionally active chromosome (TAC) proteomes [13–16]. Previous genetic approaches have demonstrated the essential role of PAPs in the regulation of PEP activity and chloroplast development. The expression of PEP-dependent genes is suppressed in mutant plants that do not express *PAPs,* resulting in defects in chloroplast development [13, 16–24]. Furthermore, studies of protein–protein interactions showed that each PAP interacts with other PAPs or PEP core proteins, indicating that the establishment of the PEP complex is a key mechanism controlling PEP activity and chloroplast development [3]. For example, pTAC3/PAP1 interacts with α core subunit of PEP [17], and pTAC14/PAP7 interacts with pTAC12/PAP5 [21]. FRUCTOKINASE-LIKE PROTEINS1 (FLN1)/PAP6 interacts with THIOREDOXIN Z (TrxZ)/PAP10 and FLN2 [22, 24], and FSD3/PAP4 interacts with FSD2/PAP9 [20]. pTAC7/PAP12 and pTAC10/PAP3 showed a broad range of interactions with other PAPs [25, 26]. A study by Pfalz et al. (2015) suggested that pTAC2/PAP2, pTAC10/PAP3, pTAC12/PAP5, and MurE/PAP11 play a key role in promoting accumulation of the fully assembled PEP complex [27].

The *Arabidopsis thaliana* genome contains three genes encoding iron superoxide dismutases, *FSD1*, *FSD2/PAP9*, and *FSD3/PAP4*; however, several lines of evidence suggest that FSD2 and FSD3 function in chloroplast development. FSD2 and FSD3 proteins localize in the chloroplasts and FSD1 localizes in the cytoplasm [20, 28]. Similar to other mutant plants in which the expression of *PAPs* is knocked out, *fsd2* and *fsd3* mutants show defects in chloroplast development, leading to a bleached-leaf phenotype. Unlike the *fsd2* and *fsd3* mutant plants, the *fsd1* mutant does not have defects in leaf color, although the expression level of *FSD1* is around 50-fold higher than that of *FSD3* [20, 29]. COPPER SUPEROXIDE DISMUTASE2 (CSD2) localizes in the chloroplasts and plays a key role in reactive oxygen species (ROS) scavenging in the chloroplasts [30, 31]. *CSD2* expression is much higher compared to other superoxide dismutase genes (around 100-fold higher than *FSD3*) [29]. However, the *csd2* mutant does not show the bleached-leaf phenotype as *PAP* mutant plants do [32, 33]. These results suggest that *FSD2* and *FSD3* have specialized functions in chloroplast development.

A single gene that contains introns can give rise to several different mRNAs via alternative splicing, thus contributing to the diversity of the proteome in eukaryotes, including plants [34]. In Arabidopsis and rice (*Oryza sativa*), only 21.7 and 19.9% of genes lack introns respectively, suggesting that alternative splicing is deeply involved in the regulation of plant development and physiology [35, 36]. Alternative splicing patterns are regulated with plant developmental stage and are frequently affected by environmental signals [37, 38]. Alternative splicing can regulate transcript abundance through RNA degradation pathways such as nonsense-mediated decay [39]. Splicing variants can encode protein isoforms with different subcellular localizations and functions, due to the insertion or deletion of functional units such as signal peptides and transmembrane (TM) domains [40, 41].

During senescence in plants, mature chloroplasts transform into gerontoplasts and photosynthetic performance decreases. PAPs play essential roles in controlling the activity of PEP, which is responsible for chloroplast development and photosynthetic activity. To understand the functions of *PAPs* in this process, we attempted to clone *PAPs* and identified *FSD3S*, a splicing variant of *FSD3/PAP4* that includes two unspliced introns*.* Unlike FSD3 proteins, which localize to the chloroplast nucleoids, FSD3S proteins have a TM domain in the C-terminal region and tended to localize to chloroplast membranes. To understand the function of FSD3S in chloroplast development, we examined the effect of *FSD3S* overexpression. Overexpression of *FSD3S* did not complement the chloroplast developmental defects in the *fsd3* mutants. In addition, the overexpression of *FSD3S* negatively regulated photosynthetic activity and chloroplast development by reducing expression of PEP-dependent genes. These results suggest FSD3 and FSD3S have different functions in chloroplast development, and FSD3S is involved in the negative regulation of PEP activity and chloroplast development.

## Results

### *FSD3S*, a splicing variant of *FSD3*

**Fig. 1:**
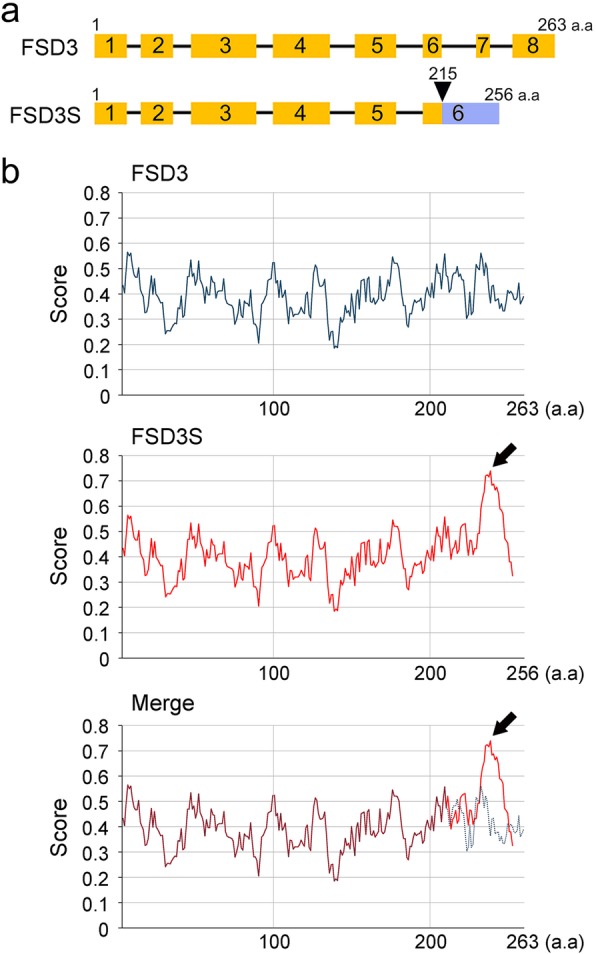
Identification of *FSD3S*, a splicing variant of *FSD3*. **a** A schematic of the *FSD3* and *FSD3S* mRNA structure. The blue box indicates the C-terminal region of FSD3S, which is composed of different amino acids from those of FSD3, and the arrowhead points to the start of this region. **b** The different hydrophobicity between FSD3 and FSD3S. A bioinformatics analysis (http://web.expasy.org/protscale/) predicted different hydrophobic properties of FSD3 and FSD3S. The black arrows indicate that the C-terminal region of FSD3S has higher hydrophobicity than that of FSD3

PAPs regulate chloroplast development by controlling activity of the PEP complex. We identified *FSD3S*, a splicing variant of *FSD3/PAP4*. *FSD3* contains 8 exons and the mature *FSD3* mRNA is generated by the removal of 7 introns from the pre-mRNA by splicing. By contrast, *FSD3S* contains only 6 exons; the 6th and 7th introns remain in the mRNA (Fig. 1a). Comparison of the predicted amino acid sequences of FSD3 and FSD3S showed that the C-terminal region of FSD3S between amino acids 215–256 was composed of different amino acids from those of FSD3 due to the presence of the 6th and 7th introns (Additional file 1: Figure S1). The stop codon of *FSD3S* is located at an earlier position (771 bp) compared to FSD3 (792 bp). Consequently, the *FSD3S* mRNA encodes a shorter protein composed of 256 amino acids, compared to 263 amino acids in FSD3.

The presence of the unspliced introns also affected the properties of FSD3S (Fig. 1b). When the hydrophobicity of FSD3 and FSD3S were predicted (http://web.expasy.org/protscale/), the C-terminal region, especially the region located between amino acids 230 and 250, differed between FSD3 and FSD3S. The C-terminal region of FSD3S displayed much higher hydrophobicity than that of FSD3, suggesting that FSD3S function might be different from that of FSD3.

### Superoxide dismutase activity of FSD3S

**Fig. 2:**
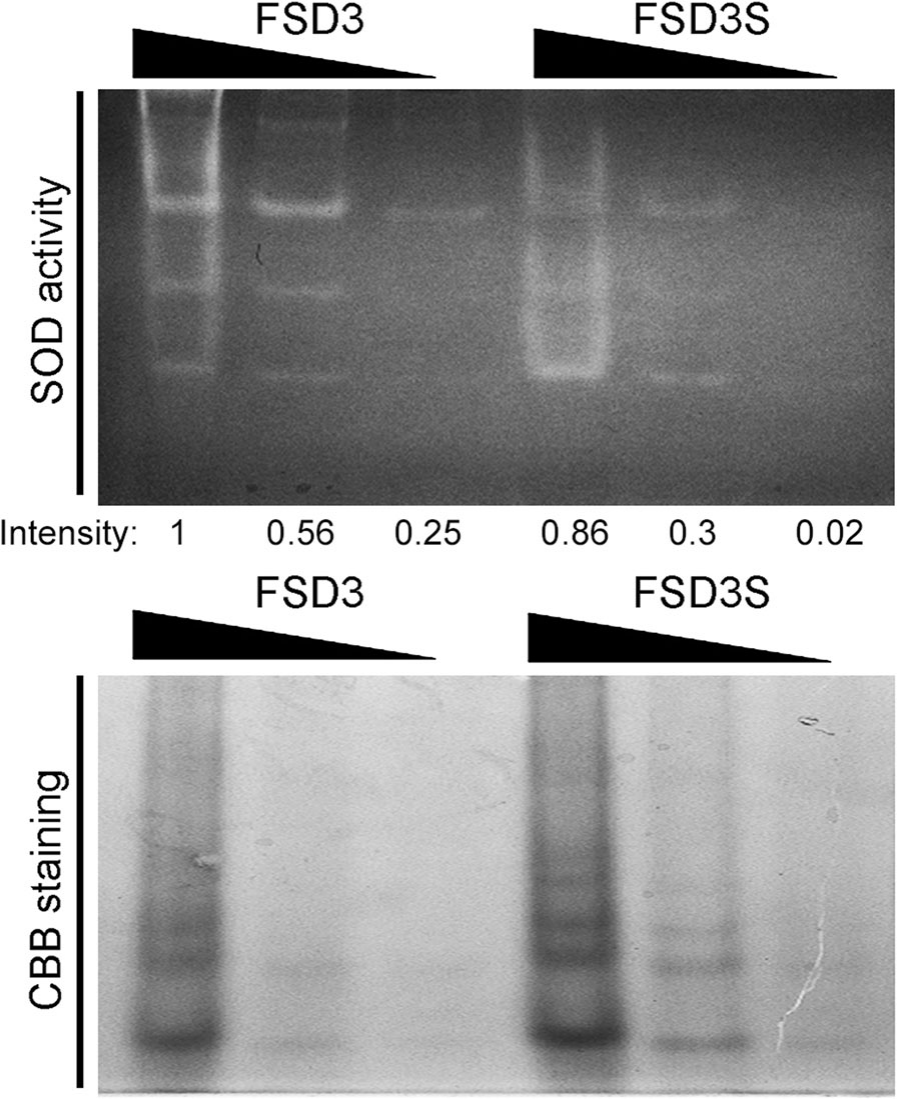
SOD activity of FSD3S proteins. To analyze SOD activity of FSD3S and FSD3, an in-gel SOD activity assay was performed using MBP-FSD3S and MBP-FSD3. MBP-fused recombinant FSD3S and FSD3 expressed in *E. coli* were purified with amylose resin. Samples containing sixteen, four, and one microgram of FSD3S or FSD3 protein were loaded on 8% native-PAGE for the test of SOD activity. Intensity indicates the relative SOD activities of FSD3 and FSD3S, which were quantified using Image J software. Coomassie brilliant blue (CBB) staining was used for loading controls

*FSD3* encodes iron superoxide dismutase. Because FSD3 and FSD3S proteins have different properties, it was expected that the superoxide dismutase (SOD) activity of FSD3S protein would be different from that of FSD3 protein. To address this, we analyzed SOD activity of FSD3 and FSD3S proteins (Fig. 2). We expressed MBP-fused recombinant FSD3 and FSD3S in *E. coli* and measured their activities with an in-gel SOD activity assay. FSD3S proteins exhibited SOD activity, and the activity of FSD3S was slightly lower or similar to that of FSD3. These observations suggested that the hydrophobic C-terminal region does not have a major effect on the SOD activity of FSD3S.

### Subcellular localization of FSD3S

**Fig. 3:**
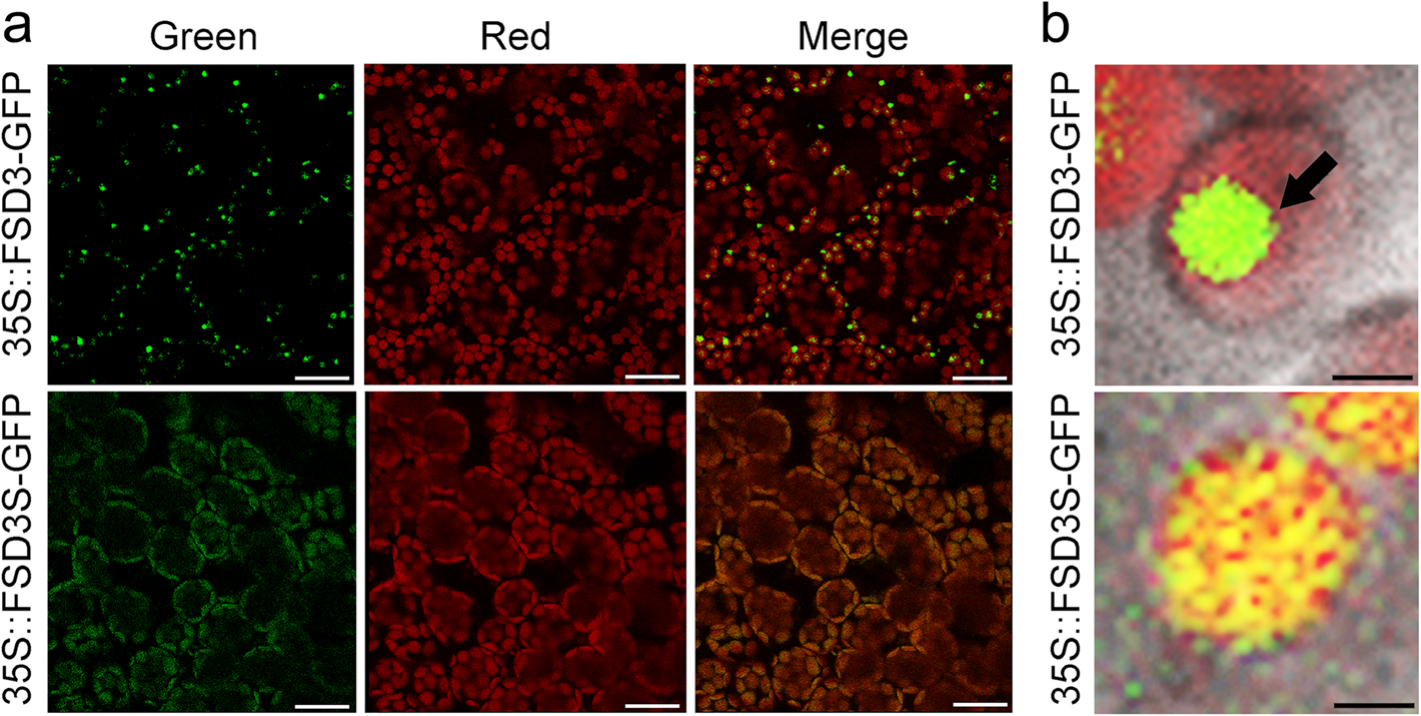
FSD3 localizes to chloroplast nucleoids, but FSD3S does not. **a** Subcellular localization of FSD3 and FSD3S was analyzed by visualizing the green fluorescent signals in the mesophyll cells of *35S::FSD3-GFP* or *35S::FSD3S-GFP* transgenic plants. **b** High magnification images showing FSD3 and FSD3S localization in chloroplasts. Green and red correspond to GFP signals and auto-fluorescence of chlorophyll, respectively. The black arrow indicates nucleoid-specific localization of FSD3. Scale bars = 20 μm in (**a**) and 2 μm in (**b**)

To test whether the hydrophobic C-terminal region of FSD3S affected its subcellular localization, we generated *35S::FSD3-GFP* and *35S::FSD3S-GFP* transgenic plants, and analyzed localization of FSD3-GFP and FSD3S-GFP by monitoring fluorescent signals in these transgenic plants. We tested at least four independent lines of *FSD3-GFP* and *FSD3S-GFP* transgenic plants. Despite some differences in the intensity of GFP signals among them, all transgenic plants exhibited green fluorescent signals in their chloroplasts. However, the subcellular localization patterns of the fluorescent signals differed between *35S::FSD3-GFP* and *35S::FSD3S-GFP* plants (Fig. 3). Consistent with a previous study by Myouga et al. [20], the fluorescent signals of FSD3 proteins appeared as dot-like structures in the chloroplasts (Fig. 3). A co-localization test using the nucleoid-associated protein PEND (PEND-CFP) [42] and FSD3 (FSD3-GFP) showed that CFP and GFP signals were in the same location in the chloroplasts (Additional file 1: Figure S2). This showed that FSD3 localizes to the nucleoids, where the PAPs and PEP complex act. However, *35S::FSD3S-GFP* did not exhibit the dot-shaped signals in chloroplasts, and the fluorescent signals of FSD3S proteins tended to be distributed throughout the chloroplasts (Fig. 3). These observations suggested that FSD3 localizes to chloroplast nucleoids but FSD3S does not. These findings suggested that the hydrophobic C-terminal region of FSD3S affects the localization of FSD3S.

### The hydrophobic C-terminal region of FSD3S contains a transmembrane domain

**Fig. 4:**
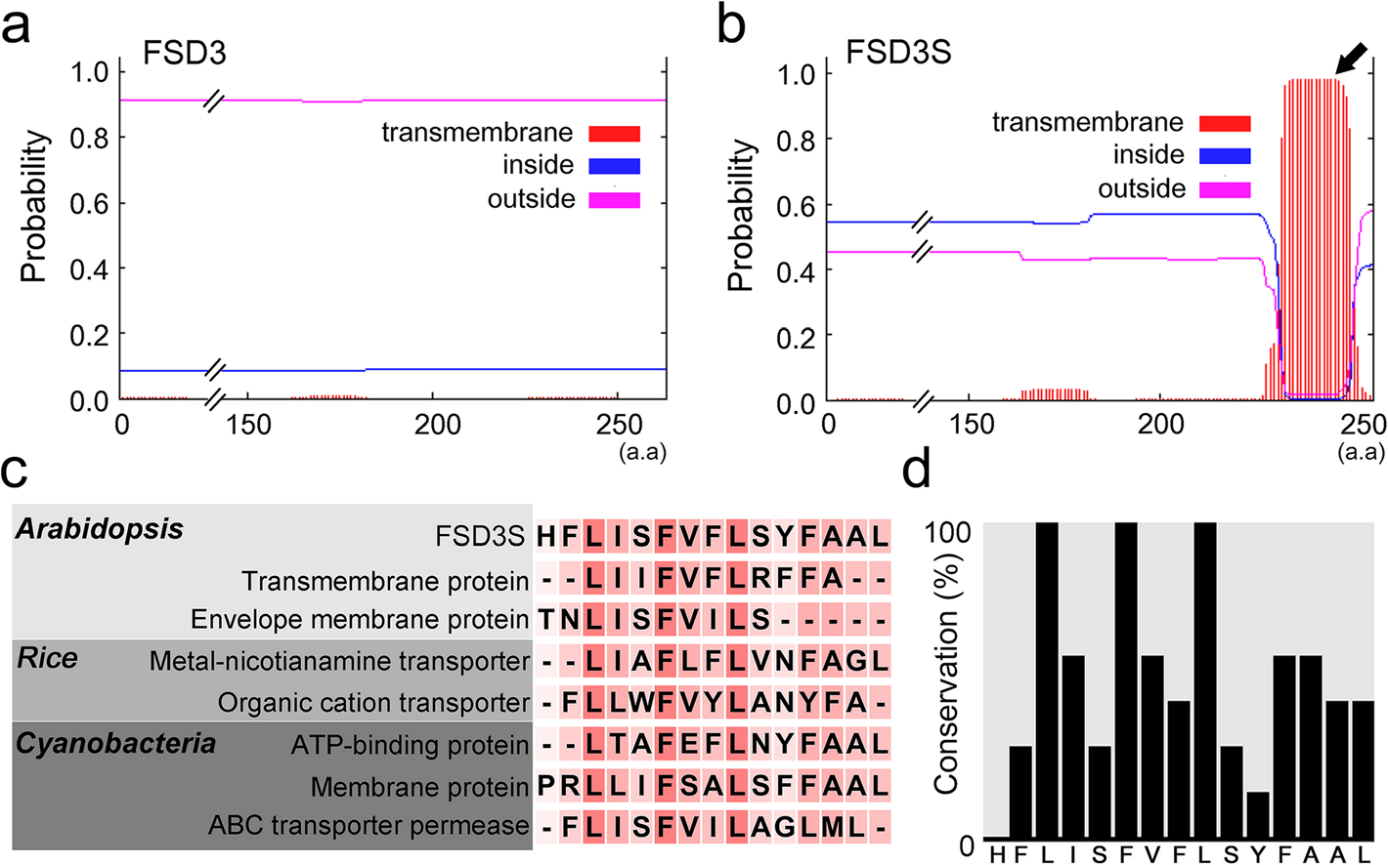
FSD3S contains a putative transmembrane helix domain. The transmembrane helix domain was predicted by bioinformatics analysis (http://www.cbs.dtu.dk/services/TMHMM/). Unlike FSD3 (**a**), the existence of a transmembrane helix domain was predicted in the C-terminal regions of FSD3S between the 231st and 245th amino acids (**b**). The black arrow indicates the transmembrane helix domain in the C-terminal regions of FSD3S. A multiple amino acid sequence alignment (**c**) and histogram (**d**) show the conservation of the transmembrane helix domains among FSD3S and other membrane or transporter proteins of Arabidopsis, rice, and cyanobacteria. Red boxes in (**c**) indicate conserved amino acids, with darker red boxes indicating a higher level of conservation

Because the N-terminal region is identical between FSD3 and FSD3S proteins but the C-terminal region differs, we expected that the hydrophobic C-terminal region of the FSD3S protein might determine the localization of FSD3S. To explore this, we performed bioinformatics analysis to identify some domains that could be responsible for the localization of FSD3S (http://www.cbs.dtu.dk/services/TMHMM/). This revealed that the hydrophobic region of FSD3S contains a putative TM helix domain, which is conserved in the TM domains of many membrane and transporter proteins of plants and microbes (Fig. 4a–d). These results suggested that FSD3S contains a TM helix domain in its C-terminal region and the TM domain affects FSD3S localization. This finding was further supported by a bioinformatics analysis of OsFSD3 in rice (Additional file 1: Figures S3 and S4). Through a protein BLAST search using the full-length amino acid sequence of Arabidopsis FSD3 (http://rice.plantbiology.msu.edu/analyses_search_blast.shtml), we identified a rice homolog of *FSD3*, *LOC_Os06g05110.1* (*OsFSD3*) and its splicing variant, *LOC_Os06g05110.3* (*OsFSD3S*). In addition, the C-terminal region of OsFSD3S protein exhibited higher hydrophobicity than that of OsFSD3, and contains a predicted TM helix domain like FSD3S. These observations indicated that FSD3S has a TM helix domain in its hydrophobic C-terminal region, and the TM domain is involved in the localization of FSD3S.

### FSD3S tends to localize at the chloroplast membrane

**Fig. 5:**
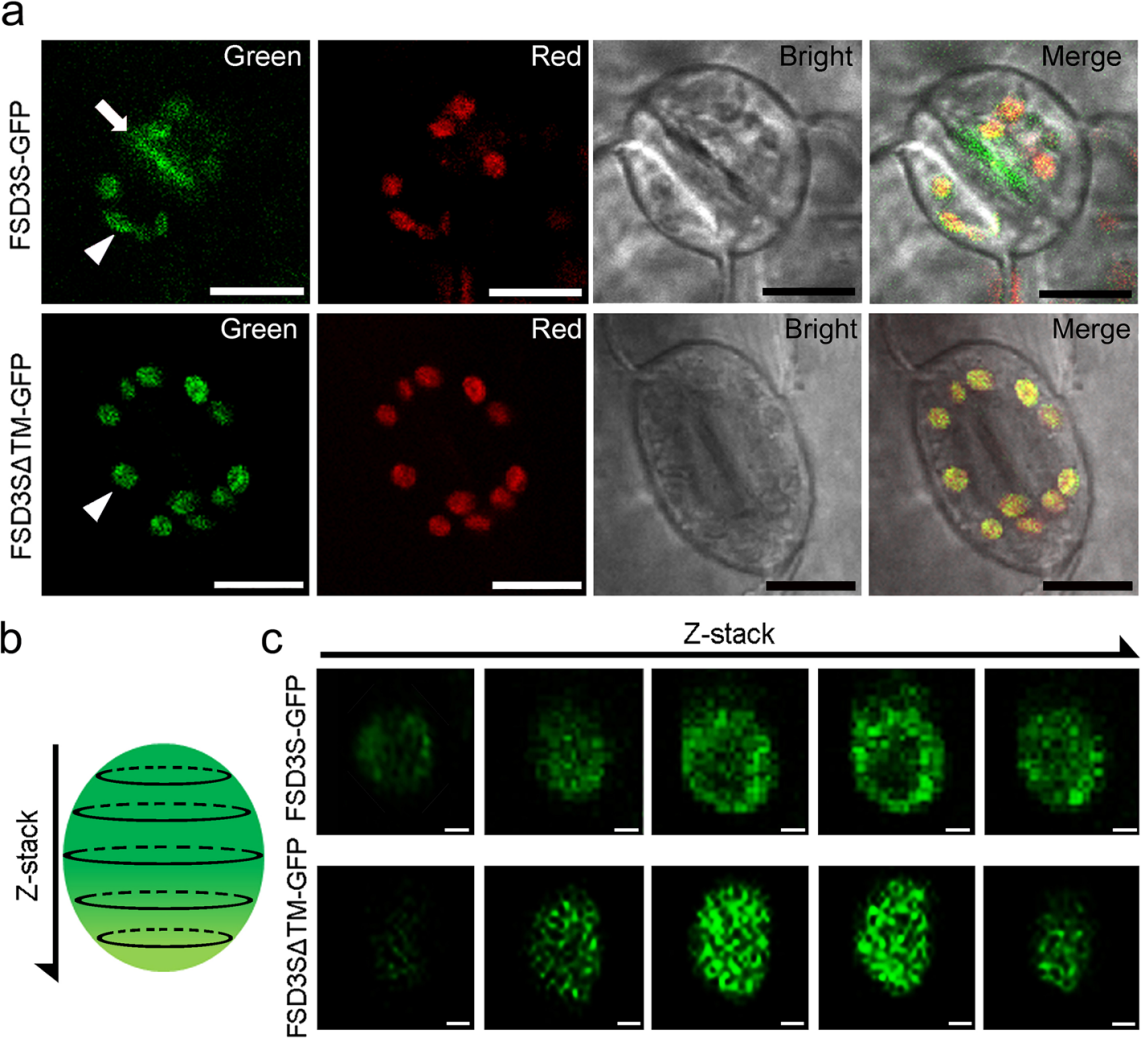
FSD3S tends to be localized to the chloroplast membrane. **a** Visualization of subcellular localization of FSD3S and FSD3SΔTM proteins lacking the transmembrane helix domain in the guard cells of *35S::FSD3S-GFP* and 35S::*FSD3SΔTM-GFP* plants. Green and red fluorescence correspond to GFP signals and auto-fluorescence of chlorophyll in chloroplasts, respectively. Bright indicates bright-field images. **b** A schematic of a series of optical sections (Z-stack) of chloroplasts using a confocal microscope. Dotted lines indicate longitudinal positions where confocal optical cross-sectioning was performed. **c** A series of z-stack images showing the fluorescent signals of FSD3S-GFP (top) and FSD3SΔTM-GFP (bottom) inside chloroplasts. Scale bars = 20 μm in (**a**) and 1 μm in (**b**)

To further understand the function of the TM domain in FSD3S localization, we generated transgenic plants expressing GFP-fused FSD3S that lacked the TM domain (FSD3SΔTM–GFP), and analyzed the fluorescent signals of FSD3SΔTM proteins in chloroplasts. Similar to the *35S::FSD3S* plants in which GFP signals do not localize to nucleoids, *35S::FSD3SΔTM–GFP* plants did not exhibit the dot-shaped signals in chloroplasts, and the fluorescent signals were diffuse and found throughout the chloroplasts (Fig. 5). However, FSD3S-GFP signals were observed along the stoma-forming membrane of the guard cells, while FSD3SΔTM-GFP signals were not (Fig. 5a; Additional file 1: Figure S5). These results suggested that the TM domain affects FSD3S localization, and FSD3S with the TM domain tends to localize to the membrane. To further explore the localization of FSD3S in chloroplasts, we performed a series of optical sections using the z-stack function of the confocal microscope. This approach revealed that the localization pattern of FSD3SΔTM is different from that of FSD3S proteins (Fig. 5b, c); FSD3S-GFP signals tended to localize to the chloroplast membranes but the FSD3SΔTM-GFP signals tended to localize diffusely throughout chloroplasts. These observations suggested that the TM domain of FSD3S promotes FSD3S localization to the chloroplast membrane. This finding was supported by western blotting assays using proteins extracted from *35S::FSD3S-GFP* and *35S::FSD3ΔTM-GFP* chloroplasts and GFP antibody (Additional file 1: Figure S6). Both FSD3 and FSD3S proteins were detected in the soluble factions. However, FSD3S proteins with the TM domain were also detected in insoluble fractions, unlike FSD3ΔTM. This suggested that the FSD3S protein with a single TM domain has an affinity to localize to the chloroplast membrane but can localize in the chloroplast stroma.

### Overexpression of *FSD3S* reduces expression of PEP-dependent genes

**Fig. 6:**
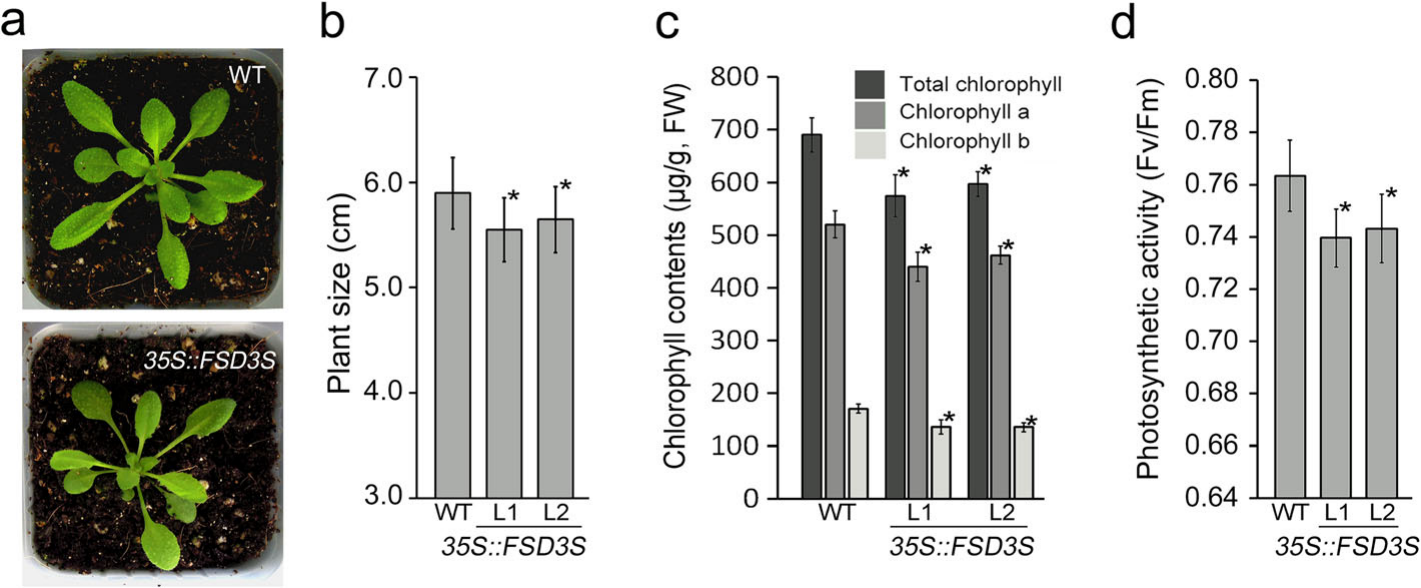
Overexpression of *FSD3S* reduces photosynthetic activity. **a** Images of wild-type and *35S::FSD3S* plants grown in soil for 5 weeks. **b** Quantification of plant size of the 5-week-old wild-type and *35S::FSD3S* plants (*n >* 22). **c** Quantification of chlorophyll contents in these plants (**c**) (the number of biological repeats, *n* = 3). **d** Measurement of photosynthetic activity (the number of leaves tested, *n* > 20). Error bars indicate SD. L1 and 2 indicate two independent lines of *35S::FSD3S* transgenic plants. Asterisks show statistically significant differences between the indicated samples (*p* value < 0.01, Student’s *t*-test)

To understand the function of *FSD3S* in chloroplast development, we generated *FSD3S-*overexpressing transgenic plants (Additional file 1: Figure S7). The transgenic plants overexpressing *FSD3S* were smaller in size than the wild-type plants grown at the same growth conditions (Fig. 6a, b). Overexpression of *FSD3S* also affected chlorophyll contents and photosynthetic activity (Fig. 6c, d). The chlorophyll contents in the *35S::FSD3S* transgenic plants were approximately 15% lower than those of the wild type grown at the same conditions. Also, the photosynthetic activity in the *FSD3S*-overexpression plants was approximately 6% lower than that of the wild-type plants. These observations indicated that *FSD3S* negatively affects chloroplast development, and the finding that expression of *FSD3S* does not rescue the *fsd3* mutant phenotype supported this (Additional file 1: Figure S8). In addition, a reduction in plant growth and photosynthetic activity was also seen in the *FSD3S-GFP* and *FSD3SΔTM*-*GFP* overexpressing plants (Additional file 1: Figure S9). This suggested that the reduction is induced by FSD3S proteins accumulated in the stroma.

**Fig. 7:**
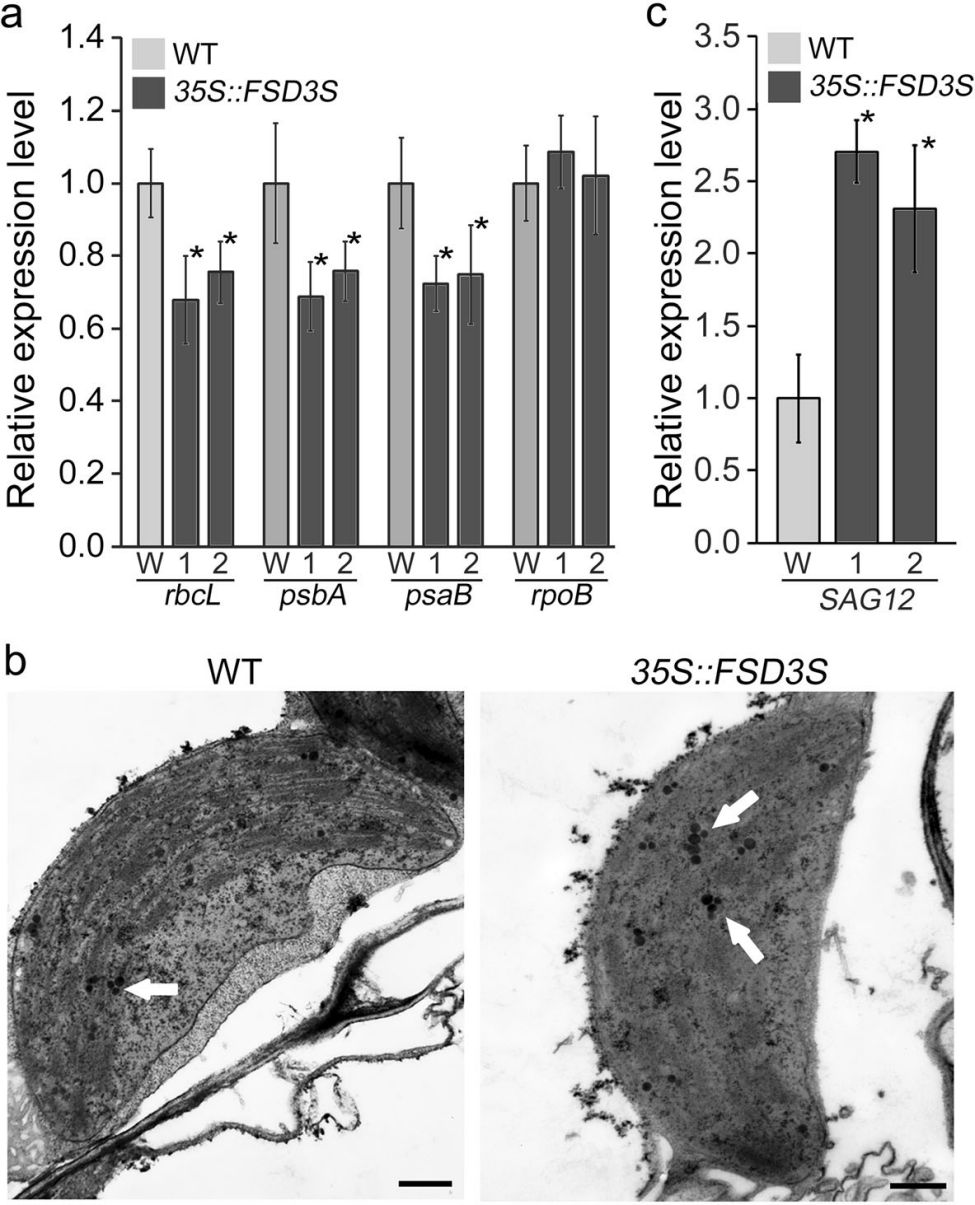
Overexpression of *FSD3S* downregulates expression of PEP-dependent genes. **a** Expression levels of PEP-dependent *rbcL*, *psbA*, and *psaB*, and NEP-dependent *rpoB* in wild-type and *35S::FSD3S* plants grown in soil for 5 weeks. **b** Chloroplast structure of 5-week-old wild-type and *35S::FSD3S* plants (line 1) were analyzed using ultra-microsectioning and TEM. **c** Expression levels of *SAG12* in these plants. Expression levels were analyzed by qRT-PCR. Data represent mean values of three biological replicates, and error bars indicate SD. Asterisks indicate statistically significant differences between the corresponding samples and their controls (*p* value < 0.01, Student’s *t*-test). W, 1 and 2 indicate Col-0 and the independent lines of the transgenic plants.

Because PAPs determine the activity of the PEP complex, which regulates expression of photosynthetic genes, it was expected that overexpression of *FSD3S* would affect the expression of photosynthetic genes. To explore FSD3S function in photosynthetic activity, we analyzed the expression of PEP-dependent and NEP-dependent chloroplast genes in *35S::FSD3S* transgenic plants (Fig. 7a). Expression of the PEP-dependent genes *rbcL, psbA,* and *psaB* were downregulated in *35S::FSD3S* transgenic plants, whereas the expression of NEP-dependent *rpoB* was similar or slightly higher compared to wild-type plants. These indicated that FSD3S negatively regulates the expression of PEP-dependent chloroplast genes, suggesting that the negative regulation of PEP-dependent photosynthetic genes is involved in the reduction of photosynthetic activity by FSD3S.

Furthermore, when we analyzed the chloroplast ultrastructure of wild-type and *35S::FSD3S* plants grown in the same growth conditions, *FSD3S-*overexpressing transgenic plants formed more plastoglobuli in their chloroplasts than wild-type plants (Fig. 7b; Additional file 1: Figure S10). Since formation of plastoglobuli is promoted along with senescence [43, 44], we hypothesized that overexpression of *FSD3S* affects senescence. Although there was not an obvious difference in senescence between wild-type and *35S::FSD3S* plants, we found that *FSD3S* overexpression promotes expression of the senescence-associated gene *SAG12* whose expression is linked to senescence [45, 46]. In 5-week-old leaves, *SAG12* expression was higher in *35S::FSD3S* than that in wild-type plants (Fig. 7c). This suggested that *FSD3S* negatively regulates chloroplast development and is involved in senescence.

This finding was supported by the expression pattern of *FSD3S* (Additional file 1: Figure S11). The transcript level of *FSD3S* was higher in 10-week-old senescent leaves than in 3-week-old young leaves, whereas the transcript level of *FSD3* was higher in 3-week-old young leaves than in 10-week-old senescent leaves. Consequently, the transcript level of *FSD3S* was approximately 20-fold lower than that of *FSD3* in young leaves, but around 2-fold lower in old leaves. These results supported the idea that FSD3 and FSD3S have different functions in chloroplast development, and suggested that *FSD3S* negatively regulates chloroplast development.

### Overexpression of *FSD3S* does not affect ROS level

**Fig. 8:**
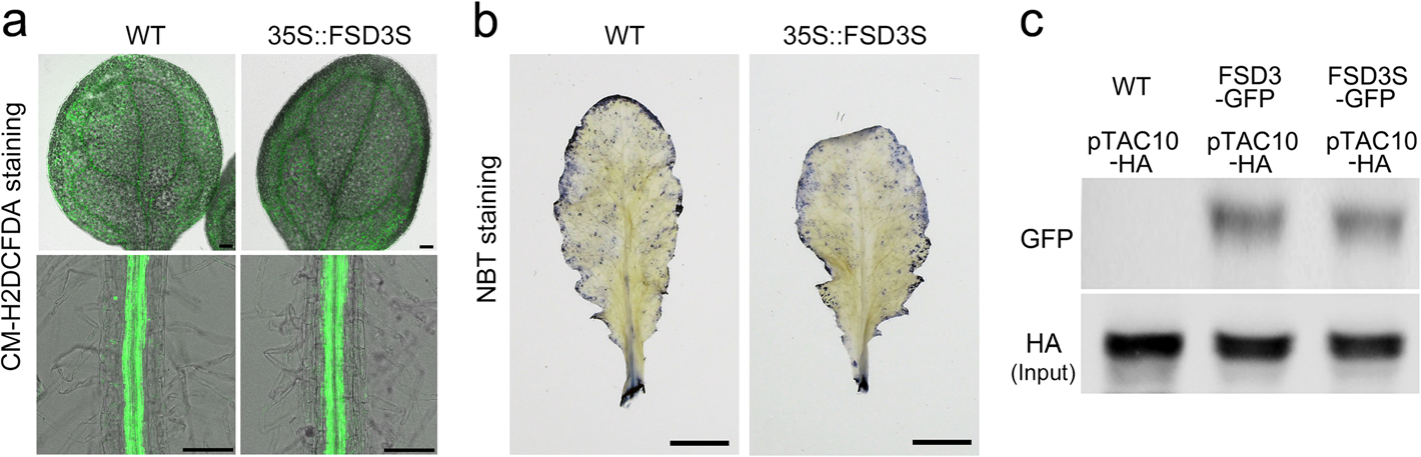
Analysis of ROS accumulation by CM-H_2_DCFDA and NBT staining. ROS accumulation was analyzed in wild-type and *35S::FSD3S* plants by CM-H_2_DCFDA (**a**) and NBT staining (**b**). CM-H_2_DCFDA staining was performed in the leaves and roots of wild-type and *35S::FSD3S* plants grown on 1/2 MS media for 2 weeks. For NBT staining, 6-week-old rosette leaves collected from the indicated plants were used. **c** Interaction between FSD3S and pTAC10. A Co-IP result showing that both FSD3 and FSD3S interact with pTAC10. Protoplasts isolated *35S::FSD3-GFP* and *35S::FSD3S-GFP* plants were transformed with *35S::pTAC10-HA* plasmid. HA antibody was used to pull down immune complex and GFP antibody was used to detect interaction of pTAC10-FSD3 or pTAC10-FSD3S. Scale bars = 100 μm in (**a**) and 2 mm in (**b**)

When the levels of reactive oxygen species (ROS) were analyzed in wild-type and *35S::FSD3S* plants by CM-H2DCFDA staining, a ROS-sensitive dye with good intracellular retention [47], we observed no obvious differences in the CM-H2DCFDA staining (Fig. 8a), suggesting that ROS levels are similar between these plants. To further test this, we stained these plants with nitroblue tetrazolium (NBT), and visualized their signals. Similar to the CM-H2DCFDA staining, the plants all showed similar intensities of the NBT staining (Fig. 8b), suggesting that overexpression of *FSD3S* does not affect ROS level, and the SOD activity of FSD3S might be not involved in the negative function of FSD3S in photosynthetic activity. Instead, we found that FSD3 and FSD3S proteins interact with pTAC10, a key PAP of PEP complex [18, 26, 27] (Fig. 8c). Because formation of PEP complex is a key process to control the activity of PEP and the transcription of photosynthesis-related genes [3], this suggested that the FSD3S-pTAC10 interaction might be involved in the negative function of FSD3S in photosynthetic activity.

## Discussion

PEP interacts with PAPs to form a functional transcription complex. A recent model proposed that PAPs play an essential role in the expression of chloroplast genes and development of chloroplasts by regulating the structural establishment of the PEP complex through PAP–PAP or PAP–PEP interactions [3, 25]. This model is supported by many molecular and genetic studies showing that each PAP interacts extensively with other PAPs or with PEP, and mutations in PAPs or PEP result in defects in chloroplast development [13, 16–19, 21–24, 48]. For example, FSD3/PAP4 interacts with other PAPs such as pTAC10/PAP3 and FSD2/PAP9, and *fsd3* mutant plants show defects in chloroplast development [20, 26].

Alternative splicing can produce two or more proteins from a single gene, and these proteins can have different functions [40]. All 12 Arabidopsis *PAP* genes contain introns, suggesting that variant PAPs might be involved in the formation of the PEP complex and chloroplast development. *pTAC12/PAP5* encodes two different protein isoforms in maize and both variant proteins assemble into the PEP complex [27], although the pTAC12/PAP5 isoforms are not produced by alternative splicing, but by post-transcriptional processes such as alternative initiation of translation or differential proteolytic cleavage. In this study, we identified *FSD3S*, a splicing variant of *FSD3*. *FSD3* and *FSD3S* encode proteins with identical N-termini, but different C-termini. We showed that the C-terminal regions of FSD3 and FSD3S are involved in their subcellular localization in chloroplasts. The plants expressing *FSD3-GFP* specifically exhibited fluorescent signals at chloroplast nucleoids where PAPs and the PEP complex act for transcription of chloroplast genes, whereas the *FSD3S-GFP* plants did not show a signal in the nucleoid. The distinct localization of FSD3S could explain why FSD3S cannot rescue the *fsd3* knock-out mutant phenotype.

In this study we also showed that overexpression of *FSD3S* reduces photosynthetic activity. The finding that overexpression of *FSD3S* downregulates the transcript levels of PEP-dependent genes involved in photosynthesis suggests that the reduction of photosynthetic activity in *FSD3S-*overexpressing plants might be caused by the reduction of PEP activity responsible for the transcription of photosynthesis genes. Our optical z-stack and western blot results showed that FSD3S proteins with a single TM domain are located in the chloroplast membrane and stroma. Because the reduction in photosynthetic activity was also induced by overexpression of *FSD3S-GFP* and *FSD3SΔTM-GFP*, these observations suggest that FSD3S accumulated in the chloroplast stroma might be responsible for the negative regulation. The molecular mechanisms underlying this process are unknown. However, it is unlikely that the SOD activity of FSD3S would be involved in this process, because there was no obvious difference in ROS levels between wild-type and *FSD3S*-overexpressing plants. Formation of the PEP complex through PEP–PAP or PAP–PAP interaction is a key process to control the activity of PEP [3]. Previous studies revealed that FSD3 interacts with other PAPs such as FSD2/PAP9 and pTAC10/PAP3 [20, 26]. This suggests that FSD3S proteins might negatively affect PEP activity by disrupting formation of the PEP complex, and the co-immunoprecipitation results showing that FSD3 and FSD3S interact with pTAC10/PAP3 partially support this idea. Further molecular and genetic approaches will expand our understanding of the mechanisms underlying this process.

## Conclusions

Since PAPs regulates expression of chloroplast genes, localization of PAPs to chloroplast nucleoids where the PEP complex acts is crucial for their function. In this study, we identified FSD3S, an isoform of FSD3. The N-terminal regions upstream of the 215th amino acid are identical between FSD3 and FSD3S, but the amino acid sequences of their C-terminal regions are completely different. The plants expressing FSD3-GFP showed specific localization of FSD3 to chloroplast nucleoids. However, FSD3S, whose C-terminal region is composed of completely different amino acid sequence from that of FSD3, did not localize to the nucleoid. Furthermore, the C-terminal region contains a TM domain and promotes FSD3S localization to the chloroplast membrane. These results indicated that the C-terminal region of FSD3 is responsible for the nucleoid-specific localization of FSD3, supporting the theory that the C-terminal region of FSD3 is essential for FSD3 function in chloroplast development. These observations provide an explanation why FSD3S cannot rescue the *fsd3* knock-out mutant phenotype. Together with the findings that overexpression of *FSD3S* reduces photosynthetic activity and expression of PEP-dependent chloroplast genes, these suggest that FSD3S negatively regulates chloroplast development.

## Methods

### Plant materials and growth conditions

*Arabidopsis thaliana* ecotype Columbia (Col-0) was used as a control in this study. The *fsd3–1* mutant (*Salk_103228*) previously described in Myouga et al. [20] was obtained from the Arabidopsis Biological Resource Center. Seeds were sterilized and plated on 1/2-strength Murashige and Skoog (1/2x MS) solid media. After 2 days of vernalization at 4 °C in darkness, plants were grown in a growth chamber with a light regime of 16/8 h (light/dark) at 22 °C. The seedlings were transferred into soil for analyses of mature plants. For the growth in continuous light or dark conditions, plants were grown in the chamber at 22 °C.

### Construction of recombinant DNA plasmids for transgenic plants

The GATEWAY system (Invitrogen) was used for construction of the recombinant DNA plasmids. For the construction of the *35S::FSD3S* construct, full-length *FSD3S* cDNA was amplified by RT-PCR from Arabidopsis total RNA. The cDNA was inserted into the pDONR221 vector (Invitrogen) by the BP reaction. The pENTRY clones were then recombined into the modified pMDC plant binary vector carrying the 35S promoter by the LR reaction. For the construction of *35S::FSD3S-GFP* and *35S::FSD3SΔTM-GFP*, each pENTRY clone containing *FSD3S*, or *FSD3SΔTM* cDNA lacking the stop codon was recombined in-frame with the 35S promoter and GFP by the LR reaction. Primer sequences are listed in Additional file 1: Table S1.

### Ultra-microsectioning and transmission electron microscopy

For the analysis of chloroplast ultrastructure, ultra-microsectioning was performed as described previously by Motohashi et al. (2001) with slight modifications [49]. Leaves collected from the indicated plants were fixed for 1 day at room temperature using fixing solution 1 (0.86 M Na-P [pH 7.2], 1% glutaraldehyde, and 1% paraformaldehyde). The samples were washed 3 times using washing solution (0.137 M Na-P [pH 7.2]) and then treated with a second round of fixation with fixing solution 2 (0.86 M Na-P [pH 7.2], 2% osmium tetroxide) for 1 h at room temperature. After being washed three times, the samples were dehydrated with an acetone gradient (25, 50, 75, and 100% in ddH_2_O (v/v)) for 1 h each and then incubated in absolute acetone overnight. The dehydrated samples were sequentially incubated in a gradient of Spurr resin (Sigma) (25, 50, 75, and 100% in acetone (v/v)) for 2 h each and in absolute Spurr resin overnight. For solidification, the samples were placed in a mold at 65 °C for 2 days. Sections (80 nm) were taken with an ultramicrotome (EM UC7, Leica). Images were captured with a transmission electron microscope (TEM), JEM1010.

### Measurement of chlorophyll content and photosynthetic activity

The chlorophyll contents were measured in 5-week-old Col-0 and *FSD3S*-*OX* as described previously by Sumanta et al. [50]. Fresh leaves (0.75 g) were homogenized with a plant tissue homogenizer with 15 ml of 95% ethanol (v/v in ddH_2_O). The homogenized samples were centrifuged at 12,000 x g for 15 min at 4 °C. The supernatants were diluted 10-fold using 95% ethanol. Chlorophyll contents were measured with a UV/visible spectrophotometer (OPTIZEN POP, Mecasys). For the measurement of photosynthetic activity in these transgenic plants, leaf disks were collected from 6th through 8th leaves of the 5-week-old Col-0 and the indicated transgenic plants. Leaf disks were kept in the dark for 30 min before measuring. The Fv/Fm values of the leaves were determined with a pulse modulation fluorometer (mini-PAM, Walz, Germany).

### In-gel SOD activity assay

The *FSD3* and *FSD3S* cDNAs were fused into the expression vector pMBP-DC using gateway system (Invitrogen). Each construct was introduced into *Escherichia coli* BL21(DE3) pLysS codon plus RIL strain, respectively. Expression of the recombinant proteins was induced by 1 mM isopropyl-β-D-thiogalactoside at 18 °C for 14 h. Crude extracts were purified with amylose resin (NEB). After washing four times with the extraction buffer (20 mM Tris-HCl [pH 7.4], 200 mM NaCl, 1 mM EDTA and 10 mM β-mercaptoethanol), elution was performed with the extraction buffer containing 10 mM maltose. The SOD activity of the proteins was analyzed as described previously by Beauchamp and Fridovich (1971) and Myouga et al. (2008) [20, 51]. Each protein was loaded onto native-PAGE. The gel was washed with ddH_2_O three times, and then incubated with NBT solution (0.1% NBT in ddH_2_O (w/v)) in the dark with gentle shaking for 15 min. After washing with ddH_2_O, the gel was immersed in riboflavin solution (0.028 mM riboflavin and 28 mM TEMED in 0.1 M potassium phosphate buffer [pH 7.0]) for 15 min and rinsed with ddH_2_O. The gel was illuminated with a white light box to initiate the photochemical reaction. Image J software was used for quantification of the SOD activity.

### Purification and fractionation of chloroplast proteins

Intact chloroplasts were isolated from wild-type, *35S::FSD3S-GFP*, and *35S::FSD3SΔTM-GFP* plants using the Minute Chloroplast Isolation Kit (Invent Biotechnologies). The chloroplasts were lysed osmotically by suspending them with 50 mM Tris-Cl [pH 7.5]. The samples were vortexed vigorously and then centrifuged at 10,000 x g for 20 min at 4 °C. The supernatants were used as the soluble fraction. For the preparation of the insoluble fraction, the remaining pellets were suspended and boiled for 5 min with 2x Laemmli Sample Buffer (Bio-Rad). The proteins were loaded into 10% SDS-polyacrylamide gels and then transferred to a polyvinylidene fluoride membrane. To detect FSD3S-GFP and FSD3SΔTM-GFP, immunoblot assay was performed using GFP polyclonal antibodies (Santa Cruz) and anti-rabbit HRP-linked secondary antibodies (Thermo Fisher). Western blot signals were detected with Amersham ECL prime (GE Healthcare).

### Quantitative RT-PCR

Quantitative RT-PCR analyses were performed using total RNA extracted from the indicated plants. Extraction of total RNA was carried out using the RNeasy plant mini-prep kit (Qiagen) and DNase was treated for 15 min according to the manufacturer’s instructions. For cDNA synthesis, 20 μL reactions were performed using 2 μg of total RNA and Superscript III reverse transcriptase (Invitrogen). For quantitative PCR, a LightCycler 480 with SYBR GREEN I Master Mix (Roche) was used. PCR and fluorescence detection were performed using a LightCycler NANO Real-Time PCR machine (Roche). PCR conditions were programmed according to the manufacturer’s instructions (initial denaturation at 95 °C for 5 min followed by 45 cycles of denaturation at 95 °C for 10 s, annealing at 60 °C for 10 s, and extension at 72 °C for 10 s). Expression levels were analyzed using three technical replicates. *AtACT2* (*At3g18780*) was used as an internal control. Three technical replicates of the qRT-PCRs were performed using three biological replicates. Primer sequence information is listed in Additional file 1: Table S1.

### Co-immunoprecipitation and co-localization assays

Protoplasts were isolated from wild-type, *35S::FSD3-GFP*, and *35S::FSD3S-GFP* plants, and transformed with the *35S::pTAC10-HA* plasmid. To construct the *35S::pTAC10-HA* plasmid*, pTAC10* cDNAs were introduced into *Bam*HI/*Not*I-digested pE2C plasmid for pTAC10-HA using the Gilson assembly system (NEB). This entry clone was inserted into the pMDC plasmid by the LR reaction. The co-immunoprecipitation assay was performed as described by Chang et al. (2017) with slight modification [26]. For the analysis of co-localization of FSD3-GFP and PEND-CFP, protoplasts isolated from *35S::FSD3-GFP* plants were used. For the construction of *35S::PEND-CFP,* a *PEND* cDNA encoding the N-terminal 88-amino acid sequence [42] was amplified and introduced into the *Bam*HI-digested pHBT-CFP plasmids. Fluorescent signals in the protoplasts were detected with a confocal microscope (STED, Leica).

### Histochemical detection of ROS by CM-H_2_DCFDA and NBT staining

To visualize ROS accumulation in the leaves, CM-H_2_DCFDA staining was performed as previously described by Foreman et al. (2003) with slight modification [47]. Two-week-old wild-type and *35S::FSD3S* plants were incubated for 1 h at 4 °C in 10 μM CM-H_2_DCFDA solution. The samples were washed with 0.1 mM KCl and 0.1 mM CaCl_2_ (pH 6.0) and then incubated for 1 h at RT. CM-H_2_DCFDA signals were visualized using a confocal microscope (STED, Leica). NBT staining was performed as described previously by Hoffmann et al. (2005) with slight modifications [52]. Mature rosette leaves collected from wild-type and *35S::FSD3S* plants grown in soil for 6 weeks were treated with NBT staining solution (6 mM NBT, 2.7 mM KCl, 1.8 mM KH_2_PO_4_, 10 mM NaH_2_PO_4_, and 137 mM NaCl [pH 7.1]). After 10 min of vacuum infiltration in the dark, the samples were exposed to light for 20 min at RT and then transferred into absolute ethanol to remove the chlorophyll. NBT staining images were captured using a digital camera, Coolpix p300 (Nikon).

## Supplementary information

**Additional file 1: Figure S1.** Nucleotide and amino acid sequences of *FSD3* and *FSD3S*. **Figure S2.** Co-localization of FSD3 and PEND. **Figure S3.** Comparison of the hydrophobicity of OsFSD3 (LOC_Os06g05110.1) and its isoform, OsFSD3S (LOC_Os06g05110.3). **Figure S4.** A putative transmembrane helix domain in OsFSD3S. **Figure S5.** Localization of FSD3S proteins. **Figure S6.** FSD3SΔTM-GFP and FSD3S-GFP proteins in chloroplasts. **Figure S7.** Expression levels of *FSD3S* in *35S::FSD3S* transgenic plants. **Figure S8.** Expression of *FSD3S* did not rescue *fsd3* mutant phenotype. **Figure S9.** Characterization of *FSD3S-GFP* and *FSD3SΔTM*-*GFP* overexpressing plants. **Figure S10.** Formation of plastoglobuli in *35S::FSD3S* transgenic plants. **Figure S11.** Changes in *FSD3* and *FSD3S* transcript levels during senescence. **Table S1.** Primers used in this study.

## Data Availability

Sequence data from this article can be found in the Arabidopsis Genome Initiative or GenBank/EMBL databases under the following accession numbers: FSD3 (At5g23310), FSD3S (KY471384), PEND (At3g52170), SAG12 (At5g45890), rpoA (AtCg00740), rpoB (AtCg00190), rbcL (AtCg00490), psbA (AtCg00020), psaB (AtCg00340) and ACT2 (At3g18780). The data and materials used in this study are available from the corresponding author on reasonable request.

## Abbreviations

FSD: Iron superoxide dismutase
PAP: Plastid-encoded RNA polymerase-associated protein
PEP: Plastid-encoded RNA polymerase
qRT-PCR: Quantitative reverse transcription polymerase chain reaction
SOD: Superoxide dismutase
