# Supplementary material for: An isoform of the plastid RNA polymerase-associated protein FSD3 negatively regulates chloroplast development
Source: BMC Plant Biol. 2019 Nov 27;19:524. doi: 10.1186/s12870-019-2128-9 (PMC6882211; doi:10.1186/s12870-019-2128-9)
Supplement: Supplementary file 1 — Additional file 1: Figure S1. Nucleotide and amino acid sequences of FSD3 and FSD3S. Figure S2. Co-localization of FSD3 and PEND. Figure S3. Comparison of the hydrophobicity of OsFSD3 (LOC_Os06g05110.1) and its isoform, OsFSD3S (LOC_Os06g05110.3). Figure S4. A putative transmembrane helix domain in OsFSD3S. Figure S5. Localization of FSD3S proteins. Figure S6. FSD3SΔTM-GFP and FSD3S-GFP proteins in chloroplasts. Figure S7. Expression levels of FSD3S in 35S::FSD3S transgenic plants. Figure S8. Expression of FSD3S did not rescue fsd3 mutant phenotype. Figure S9. Characterization of FSD3S-GFP and FSD3SΔTM-GFP overexpressing plants. Figure S10. Formation of plastoglobuli in 35S::FSD3S transgenic plants. Figure S11. Changes in FSD3 and FSD3S transcript levels during senescence. Table S1. Primers used in this study. [file 12870_2019_2128_MOESM1_ESM.pdf]

Additional File 1

Supplemental Figures

a

FSD3 CDS (792 bp)

ATGAGTTCTTGTGTTGTGACGACAAGCTGTTTCTATACAATTTCAGATTCTAGTATACGTTTGAAATC
CCCCAAGCTCCTCAATCTGAGTAACCAGCAGAGAAGACGCTCTCTTAGGTCTCGAGGTGGTTTAA
AGGTTGAAGCTTACTACGGTCTAAAGACACCTCCTTATCCACTTGATGCTTTGGAGCCGTATATGA
GTAGAAGAACACTAGAAGTGCATTGGGGAAAACACCATCGAGGTTATGTAGATAATCTGAATAAAC
AGTTAGGGAAAGATGATAGACTCTATGGATACACCATGGAAGAGCTTATCAAGGCTACATACAACA
ACGGGAATCCTTTACCCGAGTTCAACAACGCTGCACAGGTCTATAACCATGATTTCTTCTGGGAGT
CGATGCAACCTGGTGGTGGAGACACGCCTCAAAAGGGTGTTCTTGAGCAGATTGATAAGGATTTT
GGTTCTTTCACAAATTTTAGAGAAAAGTTCACTAATGCAGCTCTTACTCAGTTTGGTTCTGGATGG
GTCTGGCTTGTCTTAAAGAGGGAAGAGAGAAGGCTTGAGGTGGTCAAAACCTCAAACGCCATTAA
CCCACTCGTGTGGGACGATATTCCAATCATCTGCGTGGATGTGTGGGAGCACTCTTATTATCTGGA
CTACAAGAACGACAGGGCTAAGTATATAAACACATTTCTGAACCACTTGGTGTCGTGGAACGCTGC
CATGAGTCGGATGGCCCGTGCAGAAGCGTTTGTGAATCTTGGTGAACCCAACATCCCAATCGCTT
AA

FSD3 protein (263 a.a)

MSSCVVTTSCFYTISDSSIRLKSPKLLNLSNQQRRRSLRSRGGLKVEAYYGLKTPPYPLDALEPYMSR
RTLEVHWGKHHRGYVDNLNKQLGKDDRLYGYTMEELIKATYNNGNPLPEFNNAAQVYNHDFFWES
MQPGGGDTPQKGVLEQIDKDFGSFTNFREKFTNAALTQFGSGWVWLVLKREERRLEVVKTSNAINPL
VWDDIPIICVDVWEHSYYLDYKNDRAKYINTFLNHLVSWNAAMSRMARAEAFVNLGEPNIPIA

b

FSD3S CDS (771 bp)

ATGAGTTCTTGTGTTGTGACGACAAGCTGTTTCTATACAATTTCAGATTCTAGTATACGTTTGAAATC
CCCCAAGCTCCTCAATCTGAGTAACCAGCAGAGAAGACGCTCTCTTAGGTCTCGAGGTGGTTTAA
AGGTTGAAGCTTACTACGGTCTAAAGACACCTCCTTATCCACTTGATGCTTTGGAGCCGTATATGA
GTAGAAGAACACTAGAAGTGCATTGGGGAAAACACCATCGAGGTTATGTAGATAATCTGAATAAAC
AGTTAGGGAAAGATGATAGACTCTATGGATACACCATGGAAGAGCTTATCAAGGCTACATACAACA
ACGGGAATCCTTTACCCGAGTTCAACAACGCTGCACAGGTCTATAACCATGATTTCTTCTGGGAGT
CGATGCAACCTGGTGGTGGAGACACGCCTCAAAAGGGTGTTCTTGAGCAGATTGATAAGGATTTT
GGTTCTTTCACAAATTTTAGAGAAAAGTTCACTAATGCAGCTCTTACTCAGTTTGGTTCTGGATGG
GTCTGGCTTGTCTTAAAGAGGGAAGAGAGAAGGCTTGAGGTGGTCAAAACCTCAAACGCCATTAA
CCCACTCGTGTGGGACGATATTCCAATCATCTGCGTGGATGTGTGGGAGGTACACCATCTCCTCT
CTTCTGTCATTCTTCCAAGAAACACTTTGAAACATTTTCTAATATCTTTCGTTTTCTTATCTTACTTTG
CAGCACTCTTATTATCTGGACTACAAGGTAAAAACCTAGCTTAA

FSD3S protein (256 a.a)

MSSCVVTTSCFYTISDSSIRLKSPKLLNLSNQQRRRSLRSRGGLKVEAYYGLKTPPYPLDALEPYMSR
RTLEVHWGKHHRGYVDNLNKQLGKDDRLYGYTMEELIKATYNNGNPLPEFNNAAQVYNHDFFWES
MQPGGGDTPQKGVLEQIDKDFGSFTNFREKFTNAALTQFGSGWVWLVLKREERRLEVVKTSNAINPL
VWDDIPIICVDVWEVHHLLSSVILPRNTLKHFLISFVFLSYFAALLLSGLQGKNLA

Fig. S1 Nucleotide and amino acid sequences of *FSD3* and *FSD3S*. Nucleotide and expected amino acid sequences of *FSD3* (**a**) and *FSD3S* (**b**). The underlined amino acid sequence indicates the N-terminal region shared by FSD3 and FSD3S.

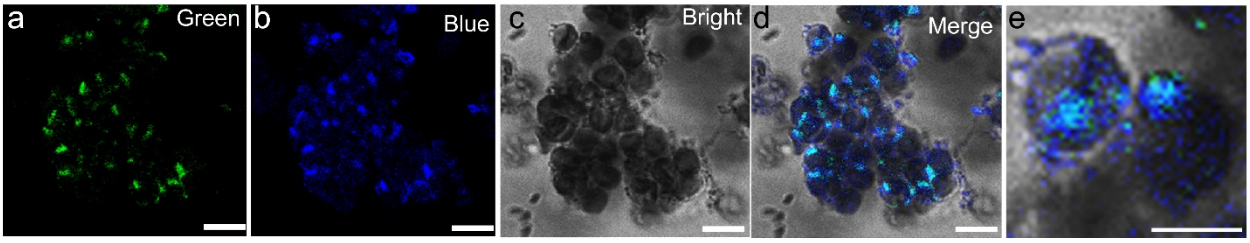

Fig. S2 Co-localization of FSD3 and PEND. Protoplasts were isolated from *35S::FSD3-GFP* plants grown in MS media for 2 weeks. The protoplasts were transformed with the *35S::PEND-CFP* plasmid. (**a-d**) Green and blue fluorescence correspond to GFP signals and CFP signals, respectively. Bright indicates bright-field images. (**e**) A high magnification image showing co-localization of FSD3 and PEND in chloroplasts. Scale bars = 50 μm in (a-d) and 25 μm in (e).

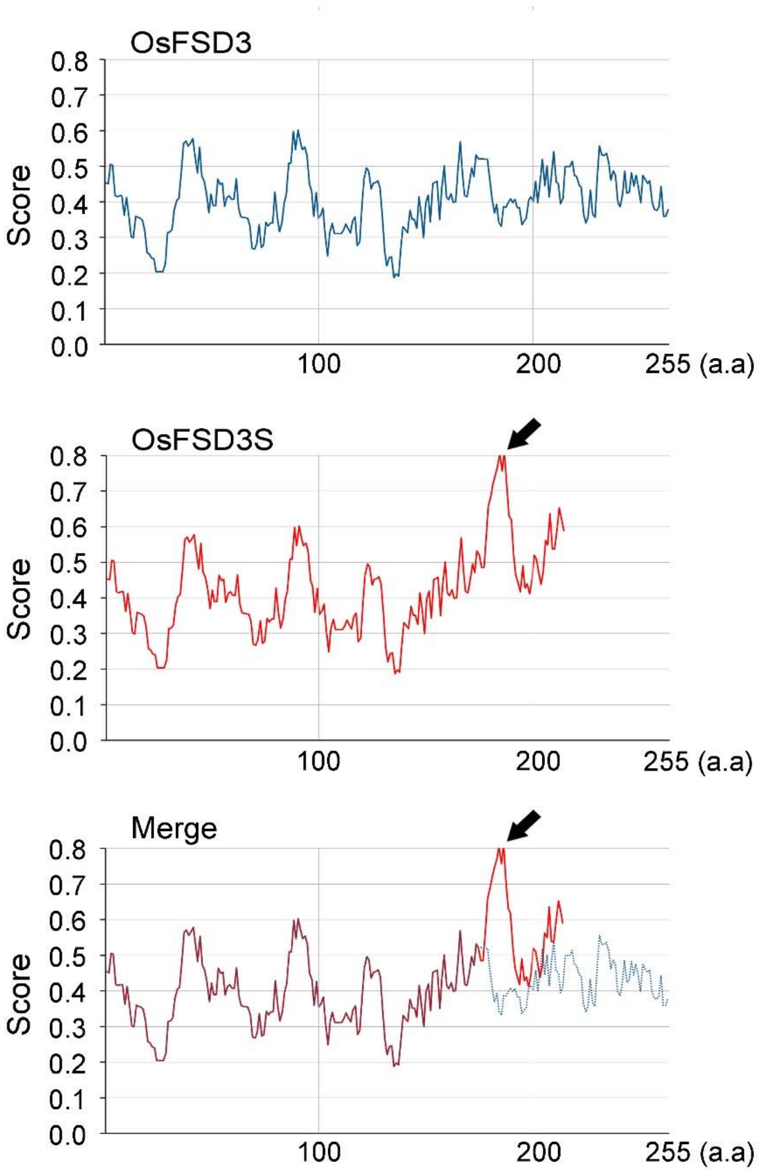

Fig. S3 Comparison of the hydrophobicity of OsFSD3 (LOC_Os06g05110.1) and its isoform, OsFSD3S (LOC_Os06g05110.3). A bioinformatics analysis predicted different hydrophobic properties of OsFSD3 and OsFSD3S (http://web.expasy.org/protscale/). The black arrows indicate that the C-terminal region of OsFSD3S has higher hydrophobicity than that of OsFSD3.

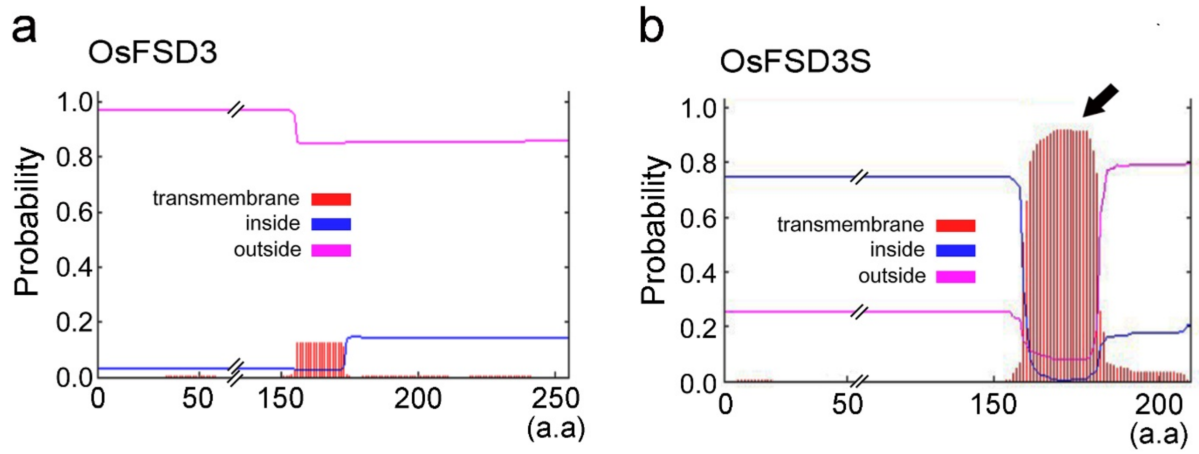

Fig. S4 A putative transmembrane helix domain in OsFSD3S. Unlike OsFSD3 (LOC_Os06g05110.1) (**a**), the existence of a transmembrane helix domain was predicted in the hydrophobic C-terminal region of OsFSD3S (LOC_Os06g05110.3) (**b**). The black arrows indicate the transmembrane helix domain.

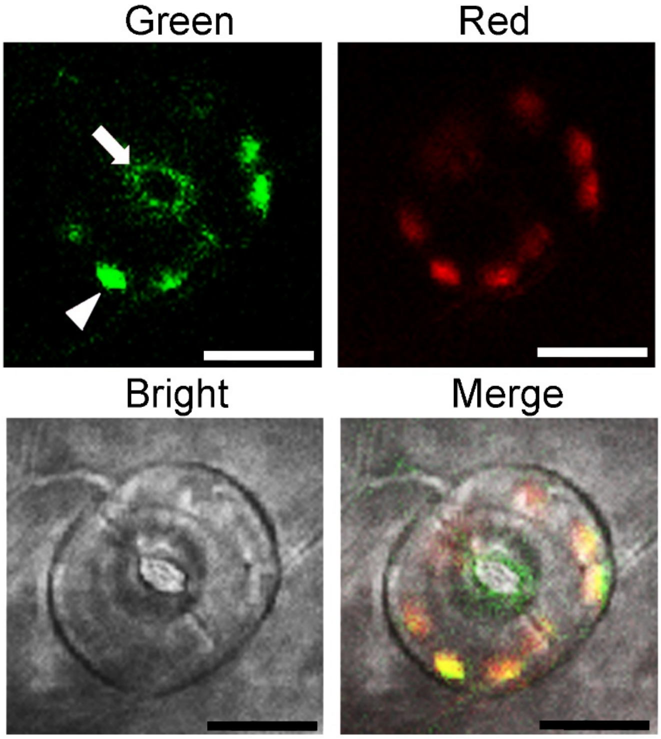

Fig. S5 Localization of FSD3S proteins. Localization of FSD3S was visualized in the guard cells of *35S::FSD3S-GFP*. The arrow and arrowhead indicate GFP signals in the stoma-forming membrane and chloroplasts, respectively. Green and red fluorescence correspond to GFP signals and auto-fluorescence of chlorophyll in chloroplasts, respectively. Bright indicates bright-field images. Scale bars = 20 μm.

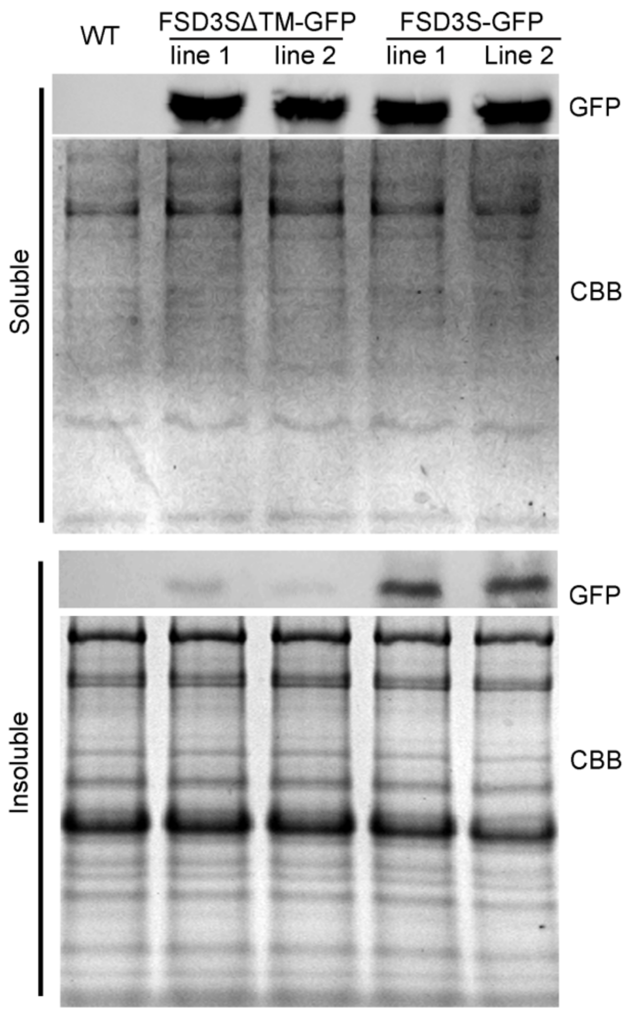

Fig. S6 FSD3SΔTM-GFP and FSD3S-GFP proteins in chloroplasts. Membrane localization of FSD3S was tested by Western blot assay. Soluble and insoluble fractions of chloroplast protein extracts were prepared from the chloroplasts isolated from 2-week-old wild-type, *35S::FSD3SΔTM-GFP*, and *35S::FSD3S-GFP* transgenic plants. These fractions were loaded on 10% SDS-PAGE. GFP antibody was used for the detection of FSD3S and FSD3SΔTM proteins. Coomassie brilliant blue (CBB) staining was used for loading controls.

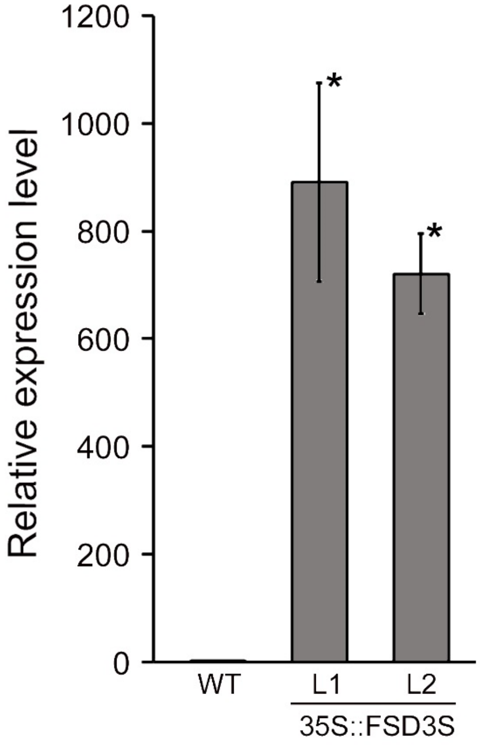

Fig. S7 Expression levels of *FSD3S* in *35S::FSD3S* transgenic plants. Expression levels of *FSD3S* were analyzed in 10-day-old *35S::FSD3S* plants by qRT-PCR. L1 and 2 indicate two independent lines of *35S::FSD3S* transgenic plants. Error bars indicate SD. Asterisks show statistically significant differences between the corresponding samples and their control (*p* value < 0.01, Student's *t*-test).

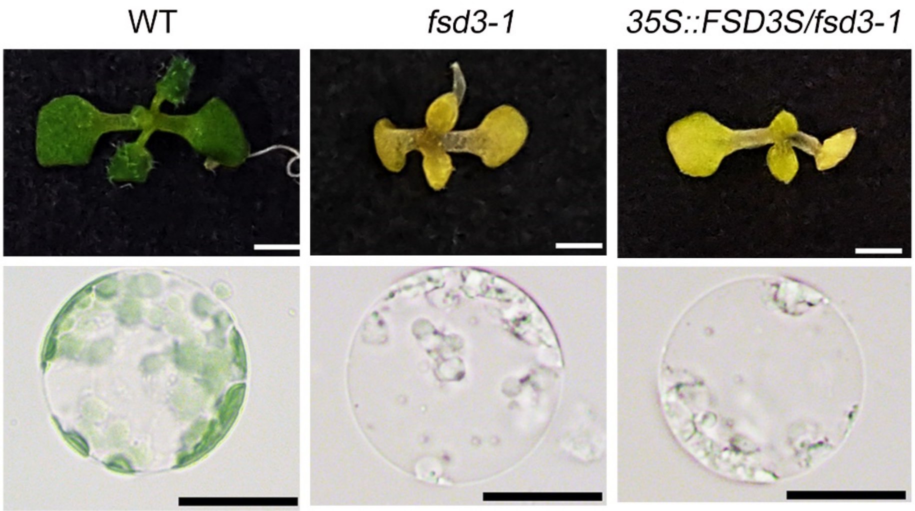

Fig. S8 Expression of *FSD3S* did not rescue *fsd3* mutant phenotype. Morphology of the 2-week-old Col-0 (WT), *fsd3-1* mutant and *fsd3-1* mutant transformed with *35S::FSD3S* plasmid (*35S::FSD3S/fsd3-1*). Scale bars = 1 mm in the whole plant images and 10 μm in the chloroplast images.

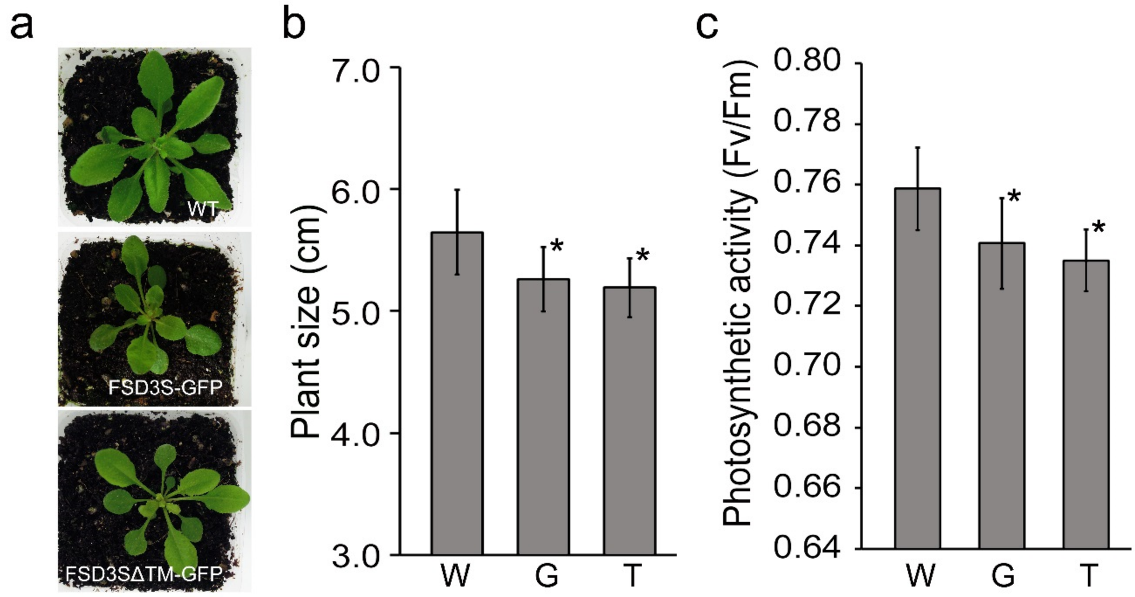

Fig. S9 Characterization of *FSD3S-GFP* and *FSD3SΔTM-GFP* overexpressing plants. (**a**) Images of wild-type, *FSD3S-GFP* (*35S::FSD3S-GFP*), and *FSD3SΔTM-GFP* (35S::*FSD3SΔTM-GFP*) plants grown in soil for 5 weeks. (**b**) Quantification of plant size of the wild-type (W) and *FSD3S-GFP* (G) and *FSD3SΔTM-GFP* (T) plants (*n*>15). (**c**) Measurement of photosynthetic activity (the number of leaves tested, *n*>15). Error bars indicate SD. Asterisks show statistically significant differences between the corresponding samples and their control (p value < 0.01, Student's *t*-test).

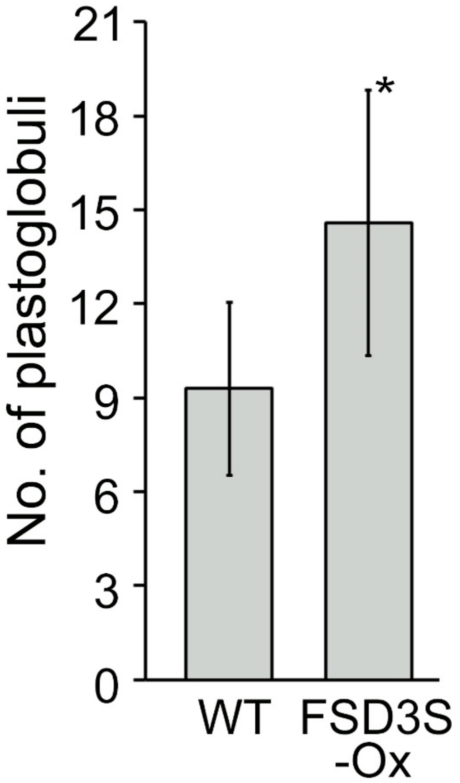

Fig. S10 Formation of plastoglobuli in *35S::FSD3S* transgenic plants. Quantification of plastoglobuli formation in the chloroplasts of wild-type (Col-0) and *35S::FSD3S* plants (*FSD3S-Ox*) grown at the same growth condition for 5 weeks (n=7). Error bars indicate SD. Asterisks show statistically significant differences between the corresponding samples and their control (p value < 0.02, Student's t-test).

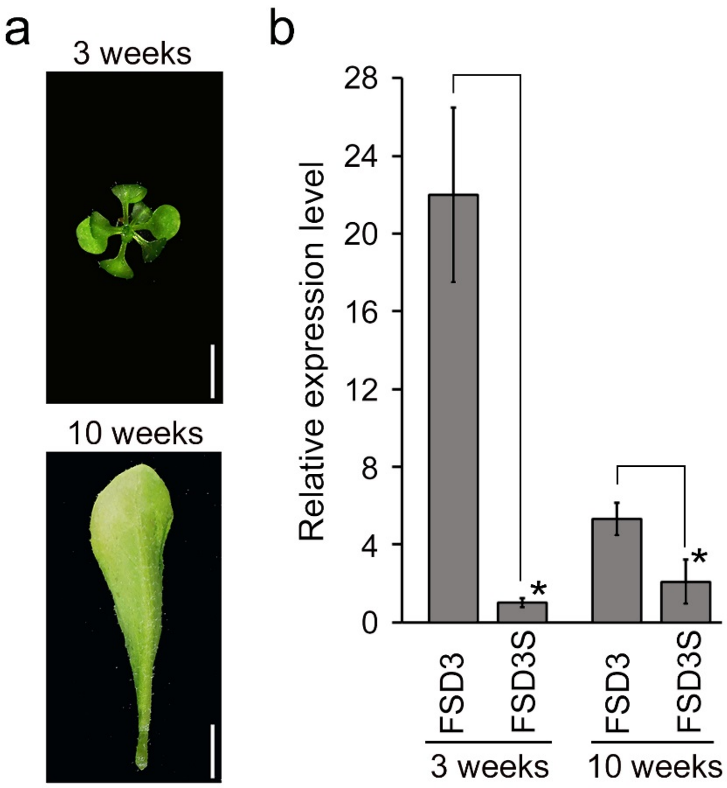

Fig. S11 Changes in *FSD3* and *FSD3S* transcript levels during senescence. Morphology of 3-week-old and 10-week-old leaves (a), and a qRT-PCR result showing transcript levels of *FSD3* and *FSD3S* in the leaves (b). Error bars indicate SD. Asterisks show statistically significant differences between the indicated samples (p value < 0.01, Student's t-test). Scale bars = 0.5 cm.

Table S1. Primers used in this study

Name	Sequence	Purpose
FSD3 geno-5	GCCTTCTAGCTCACGGCTTG	fsd3 genotype
FSD3 geno-3	GCAGCGTTGTTGAACTCGGG	fsd3 genotype
FSD3-5	CTGGACTACAAGAACGACAG	qRT-PCR
FSD3S-5	TCCGGTTGCTAGAACGACAG	qRT-PCR
FSD3 and 3S-3	GCGATTGGGATGTTGGGTTC	qRT-PCR
rpoB-5	GAACATCTGCAATACCCGG	qRT-PCR
rpoB-3	GTTCTACCAATTGATATGTTTCC	qRT-PCR
rbcL-5	GCTGCTGAATCTTCTACTGG	qRT-PCR
rbcL-3	GAGTTTCTTCTCCTGGAACG	qRT-PCR
psbA-5	GTCCTTGGATTGCTGTTGC	qRT-PCR
psbA-3	GAGATTCCTAGAGGCATACC	qRT-PCR
SAG12-5	GATGTCAAGGATAAACAAGGAC	qRT-PCR
SAG12-3	CAATCCCACACAAACATACAC	qRT-PCR
ATC2-5	CTTGCACCAAGCAGCATGAA	qRT-PCR
ATC2-3	CCGATCCAGACACTGTACTTCC	qRT-PCR
FSD3 BP-5	GGGGACAAGTTTGTACAAAAAAGCAGGCTCCATGAGTTCTTG TGTTGTG	GFP, Ox
FSD3S BP-3	GGGGACCACTTTGTACAAGAAAGCTGGGTCTTAAGCTAGGTT TTTACCT	OX
FSD3 BP-3 nostop	GGGGACCACTTTGTACAAGAAAGCTGGGTCAGCGATTGGGAT GTTGGGTTC	GFP and GST fusion
FSD3S BP-3 nostop	GGGGACCACTTTGTACAAGAAAGCTGGGTCAGCTAGGTTTTT ACCTTGTAG	GFP and GST fusion
FSD3SΔTM BP-3 nostop	GGGGACCACTTTGTACAAGAAAGCTGGGTCAAAATGTTTCAA AGTGTTTCTTG	GFP and GST fusion
PEND fusion-5	CCCCTTGCTCCGTGGATCCATGCACTCTCTTAAGACTACTTGC	CFP fusion
PEND fusion-3 no stop	CCTCGCCCTTGCTCACGGATCCACCAGGACCAAGGACTC	CFP fusion
CFP fusion-5	GTGAGCAAGGGCGAGGAG	CFP fusion
CFP fusion-3	CCAAATGTTTGAACGATCTGCAGTCACTTGTACAGCTCGTCCA TG	CFP fusion
pTAC10 HA-5	GGAACCAATTCAGTCGACTGGATCCATGCAGATTTGCCAAAC CAAG	Co-IP
pTAC10 HA-3	CAGGCCTCCGCTTGCGGCCGCGTCTGTCAAGACTTGAGTAC	Co-IP
